# Anomalies in T Cell Function Are Associated With Individuals at Risk of *Mycobacterium abscessus* Complex Infection

**DOI:** 10.3389/fimmu.2018.01319

**Published:** 2018-06-11

**Authors:** Viviana P. Lutzky, Champa N. Ratnatunga, Daniel J. Smith, Andreas Kupz, Denise L. Doolan, David W. Reid, Rachel M. Thomson, Scott C. Bell, John J. Miles

**Affiliations:** ^1^QIMR Berghofer Medical Research Institute, Brisbane, QLD, Australia; ^2^Centre for Biodiscovery and Molecular Development of Therapeutics, Centre for Biosecurity and Tropical Infectious Diseases, AITHM, James Cook University, Cairns, QLD, Australia; ^3^Faculty of Medicine, University of Queensland, Brisbane, QLD, Australia; ^4^Department of Thoracic Medicine, The Prince Charles Hospital, Brisbane, QLD, Australia; ^5^Gallipoli Medical Research Institute, Brisbane, QLD, Australia; ^6^Institute of Infection and Immunity, Cardiff University School of Medicine, Cardiff, United Kingdom

**Keywords:** non-tuberculous mycobacteria, cystic fibrosis, immunoprofiling, pulmonary non-tuberculous mycobacteria infection, T cells

## Abstract

The increasing global incidence and prevalence of non-tuberculous mycobacteria (NTM) infection is of growing concern. New evidence of person-to-person transmission of multidrug-resistant NTM adds to the global concern. The reason why certain individuals are at risk of NTM infections is unknown. Using high definition flow cytometry, we studied the immune profiles of two groups that are at risk of *Mycobacterium abscessus* complex infection and matched controls. The first group was cystic fibrosis (CF) patients and the second group was elderly individuals. CF individuals with active *M. abscessus* complex infection or a history of *M. abscessus* complex infection exhibited a unique surface T cell phenotype with a marked global deficiency in TNFα production during mitogen stimulation. Importantly, immune-based signatures were identified that appeared to predict at baseline the subset of CF individuals who were at risk of *M. abscessus* complex infection. In contrast, elderly individuals with *M. abscessus* complex infection exhibited a separate T cell phenotype underlined by the presence of exhaustion markers and dysregulation in type 1 cytokine release during mitogen stimulation. Collectively, these data suggest an association between T cell signatures and individuals at risk of *M. abscessus* complex infection, however, validation of these immune anomalies as robust biomarkers will require analysis on larger patient cohorts.

## Introduction

Pulmonary infection caused by non-tuberculous mycobacteria (NTM) is an emerging threat with serious public health consequences. Mortality rates of 10–40% due to lung disease caused by these lesser known “cousins” of *Mycobacterium tuberculosis* (TB) have been increasingly reported in the developed world (1–4). The prolonged treatment regimens lasting months to years and increasing antibiotic resistance to front-line antibiotics make these pathogens difficult and expensive infections to treat. Over 180 species of NTM are known to cause disease in humans of which the *Mycobacterium avium* complex (MAC) and the *Mycobacterium abscessus* complex (MABS) are of dominant clinical interest (5). These species account for over 80% of NTM disease worldwide and are among the most common causative agents for NTM lung disease (6). The global increase in disease prevalence over the past 10–15 years has led to an increased focus on patient-oriented research (7, 8).

The emergence and spread of human transmissible clones of MABS has been recently reported (9) and is the first evidence of person-to-person transmission of NTM that were, up until to now, considered environmentally acquired by susceptible individuals. MABS infection is associated with rapid decline in lung function and extensive lung damage which can be life threatening, particularly in patients already compromised with respiratory problems such as those with cystic fibrosis (CF). Multi-drug resistance (MDR) of these pathogens contributes to prolonged and difficult treatment regimens and high relapse rates, both of which lead to increased morbidity/mortality and escalating treatment costs in a group of patients who are already highly susceptible to opportunistic infections. The presence of MABS is an absolute contradiction to lung transplantation (10–13).

Non-tuberculous mycobacteria infections are also a growing health concern among the elderly population. Pre-existing lung diseases, such as chronic obstructive pulmonary disease and bronchiectasis are known risk factors for developing NTM infection as are lung malignancies, immune modulatory treatments, and HIV/AIDS (7, 14). The worldwide increase in NTM infections in apparently immunocompetent middle aged to elderly patients, in the setting of an aging population contributes to an increased population of susceptible individuals at-risk of developing NTM infection.

Delineating immune function in NTM infection is of fundamental interest in order to understand how and why these infections: (i) occur in specific at-risk populations; (ii) progress in some patients and; (iii) resolve in others. The importance of Th1-type cell-mediated immunity in anti-mycobacterial immunity is well established. Low production of the Th1 cytokines IFNγ and TNFα and more recently, low production of IL-17 and IL-10 have been associated with NTM infection (15–21).

Cytotoxic T-lymphocyte-associated protein 4 (CTLA-4), programmed cell death protein 1 (PD-1), and T-cell immunoglobulin domain and mucin domain 3 (TIM-3) are negative regulatory check points that are important for T cell tolerance and regulation during the immune response. Widely known for their use as targets in cancer immunotherapy (22), these immune checkpoints have also been shown to play an important role in T cell exhaustion during chronic infections such as TB (23–27). The role of these molecules in NTM infection has yet to be explored. Information on T cell “quality” in terms of cytokine production is also lacking. Polyfunctional T cells are known to determine pathogenesis and disease progression of TB and other infectious and immune-related diseases (26, 28–30).

In order to discover new targets for therapeutic intervention and rational vaccine design, an improved understanding of the molecular and cellular host defense mechanisms that provide protective immunity toward NTM is required. The present study comprehensively characterized the immune profile of NTM patients by performing high-dimensional flow cytometry-based analysis in two cohorts of NTM patients. The first group was CF patients and the second group was immunocompetent middle aged to elderly patients with MABS infection. We show across both groups abnormalities in global T cell function that associate with individuals at risk of infection.

## Patients and Methods

### Patient Cohorts

Two patient cohorts were studied. The CF patient cohort (*n* = 24) included three groups of patients; (i) CF patients with active pulmonary MABS infection (defined as MABS identified by at least one positive culture within a 12-month period) *n* = 6 (CF^Act^) and one with MAC infection; (ii) patients who had a previous diagnosis of MABS infection who had undergone treatment and were now in remission as defined by at least six clear sputum samples (smear and culture negative over a 1-year period; CF^Past^ *n* = 8) and; (iii) patients who were confirmed as having chronic *Pseudomonas aeruginosa* (Pa) infection by the Leeds criteria (31), but with no history of or current NTM infection were included as a “within-disease” control group (CF^Controls^ *n* = 9). Five of the six patients with active MABS infection had chronic Pa infection and one had intermittently positive sputum cultures (≤50% of serial sputum cultures positive) for Pa. Sex, age, and demographic-matched healthy controls were recruited from unrelated adult volunteers (HC^A^ *n* = 10). The second patient cohort was elderly patients diagnosed with active NTM (NTM^Act^ *n* = 10) all of whom had confirmed MABS infection. Healthy controls (HC^B^) sex, age, and demographic-matched to the NTM^Act^ patients were recruited from healthy adult volunteers (*n* = 10). All samples were obtained with written consent and all protocols were approved by the Human Research Ethics Committees of the QIMR Berghofer Medical Research Institute, The Prince Charles Hospital, and Greenslopes Private Hospital, Australia.

### Blood Samples and Processing

Peripheral blood mononuclear cells (PBMC) were separated from venous blood by Ficoll–Paque PLUS (GE Health) density gradient method and were cryopreserved in R10 medium (RPMI-1640 containing 10% fetal bovine serum (FBS) with 100 U/ml penicillin and 100 µg/ml streptomycin) supplemented with 10% DMSO (Sigma-Aldrich). Thawed cells were rested overnight in R10 medium at 37°C. Cells were then stained for viability, counted, and aliquoted into three 96-well plate. Cells in plate one were resuspended in staining buffer (PBS with 2% FBS) and stained for direct *ex vivo* flow cytometric analysis. Cells in plate two and three were split into two aliquots and one aliquot was activated with PMA ionomycin (PMA/I) (Ebioscience) at 1× final concentration in the presence of Brefeldin A 1 μg/ml (Ebioscience) and Monensin 0.1 μg/μl (Ebioscience) for 6 h at 37°C. The other aliquot was incubated in R10 without PMA/I as an *ex vivo* baseline control.

### Flow Cytometric Analysis

The cells in plate one were stained for surface markers delineating major immune cell lineages and evaluated for TIM-3 expression. Panel one included surface markers αCD4-FITC (BD), αCD8-Percp-Cy5.5 (Biolegend), αCD16-PECY7 (BD), αCD19-BV421 (BD), αCD14-APC (BD), and αTIM-3-PE (R&D Systems). Cells in plate two were stained with panel 2 which included surface markers αCD4-FITC (BD), αCD8-Percp Cy5.5 (Biolegend), activation marker αCD25-PE (BD), and exhaustion marker αPD-1-BV605 (BD). Staining for intracellular exhaustion marker αCLTA-4-BV421 (BD) and nuclear transcription factor αFOXP3-APC was performed after fixation and permeabilization on ice with FOXP3 Permeabilization kit (Ebioscience) according to manufacturers’ instructions. Plate three cells were activated with PMA/I and incubated with αCD107a-FITC (BD) during activation. Cells were then washed and resuspended in staining buffer and surface staining was performed with panel 3; αCD3-PECY7 (BD), αCD4-BV711 (BD), and αCD8-Percp-Cy5.5 (BD) markers. Intracellular cytokines were stained with αINFγ-AlexaFlour700 (BD), αTNFα-APC (BD), and αIL-2-PE (BD) after fixation and permeabilization with Fix/Perm buffer kit (BD) for intracellular staining. Stained samples were run on a BD LSR Fortessa 4 laser cytometer (BD). Sample acquisition was performed on BD FACSDiva 8.0 (BD) and data were analyzed with FlowJo v10 (TreeStar) and Cytobank^1^ for viSNE analysis (32).

### Statistical Analysis

Statistical analysis was performed with SPSS 22, Graphpad PRISM (v6.05), and Gmine^2^ (33). Comparison of means over multiple groups was performed using one-way ANOVA tests with Tukeys *post-hoc* comparisons, while comparisons between two groups were performed using unpaired *t*-tests or Wilcoxon rank test. Hierarchical clustering and biomarker identification was performed in Gmine. Stepwise regression was performed to identify variables associated with NTM disease. The patient in CF^Act^ group with active MAC infection was excluded from these analyses. However, the patients’ data point was included in scatter plots as a blue circle to visually demonstrate how active MAC infection compares to active MABS infection. Polyfunctionality analysis was performed using Pestle and SPICE V5^3^ (34).

## Results

### Distinct T Cell Phenotype in CF Patients Susceptible to MABS Infection

We first investigated the phenotypic and functional immune profiles of PBMCs in CF patients to probe for functional deficiencies that could underlie predisposition to NTM infection. Cohorts were categorized as CF with active NTM infection (CF^Act^), CF with a past history of NTM infection (CF^Past^), CF with chronic Pa infection (and no history of NTM infection CF^Control^) and healthy controls (HC^A^), all matched in both age (ANOVA *P* = 0.350) and gender distribution (Chi-square *P* = 0.445). Demographic and clinical characteristics of patient groups are shown in Table 1. All patients had either active or past MABS infection with the exception of one patient who had active MAC infection.

**Table 1:** Table shows demographic of cystic fibrosis (CF) cohorts and elderly patient group.

	CF^Act^	CF^Past^	CF^Controls^	NTM^Act^
Mean age (SD)	32.6 (13.6)	34.9 (10.4)	33.1 (8.0)	75.6 (9.23)
Male:female	06:01	06:02	06:03	03:07
Non-tuberculous mycobacteria (NTM) infection at time of sample	MABS (6) MAC (1)^a^	None	None	MABS (10)
**History of NTM infection**				
MABS	0	8	0	0
MAC	3^b^	0	0	3^c^
**Other infections**				
*Pseudomonas aeruginosa*	5	8	9	4
*Aspergillus* spp	0	0	0	1
*Burkholderia* spp^d^	0	1	1	0
**Lung function**				
>70% FEV1	4	1	3	8
30–70% FEV1	3	6	5	2
<30% FEV1	0	1	1	0
**Radiographic features**				
Bronchiectasis	7	8	9	10

*CF^Act^-CF patients with active NTM infection. CF^Past^-CF patients with past NTM infection. Both groups of patients had a history of P. aeruginosa infection. CF^Controls^-control CF patients with chronic P. aeruginosa infection. NTM^Act^-elderly patients with active NTM infection. N/A, not applicable; MABS, Mycobacterium abscessus complex; MAC, Mycobacterium avium complex*.

**Figure 1:**
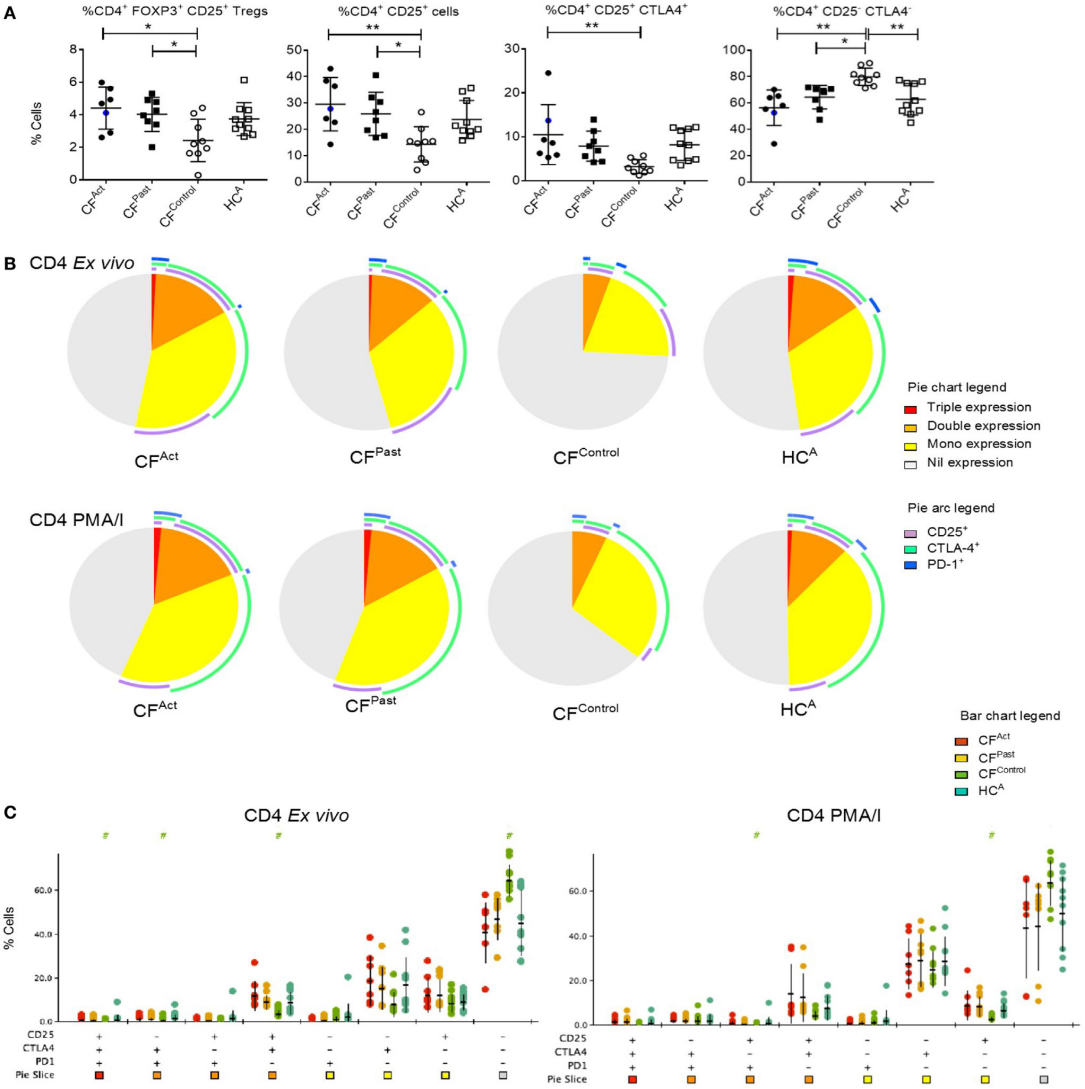
Divergent T cell activation and exhaustion profiles in cystic fibrosis (CF) patients based on non-tuberculous mycobacteria (NTM) infection status. **(A)** Flow cytometric analysis of *ex vivo* CD4^+^ T cells show significant differences in Treg percentages and marker expression between patient groups. Significantly more Tregs were seen in both CF^Act^ and CF^Past^ groups compared to CF^Contro1^ (one-way ANOVA with *post-hoc* testing *P* = 0.013 and *P* = 0.042, respectively). Significantly higher CD25^+^ CD4^+^ T cells were observed in both CF^Act^ and CF^Past^ groups compared to CF^Contro1^ group (*P* = 0.0056 and *P* = 0.037, respectively). CD25 cytotoxic T-lymphocyte-associated protein 4 (CTLA-4) double-positive T cells were significantly higher in CF^Act^ than in CF^Control^ (*P* = 0.019). A reciprocal reduction in CD25 CTLA-4 double-negative CD4^+^ T cells were seen in CF^Act^, CF^Past^, and HC^A^ groups compared to CF^Control^ (*P* = 0.001, *P* = 0.027, and *P* = 0.008, respectively). CF^Act^ patient with active *Mycobacterium avium* complex (MAC) infection is shown as a blue circle in scatter plots. This data point was not included in the ANOVA analysis but is shown here to demonstrate activation and exhaustion profile of a patient with an active NTM infection that is not MABS **(B)** Immune marker profiling of CD4^+^ T cells by SPICE showed differences in *ex vivo* phenotype in the CF^Contro1^ group compared to CF^Act^ (*P* = 0.0002), CF^Past^ (*P* = 0.0002), and HC^A^ (*P* = 0.005). PMA/I stimulation resulted in minor changes in maker profile with the CF^Control^ profile still being significantly different to CF^Act^ (*P* = 0.025) and CF^Past^(*P* > = 0.018) though the difference with HC^A^was reduced (*P* = 0.057). **(C)** SPICE dot plots show expression levels of all combinations of markers CD25, CTLA-4, and programmed cell death protein 1 in CD4^+^ T cells both *ex vivo* and after PMA/I stimulation in CF patient and control groups. Groups with significantly different expression compared to HC^A^ (Wilcoxon rank test *P* < 0.05) are indicated with # symbol. Symbol color indicates significantly different group.

**Figure 2:**
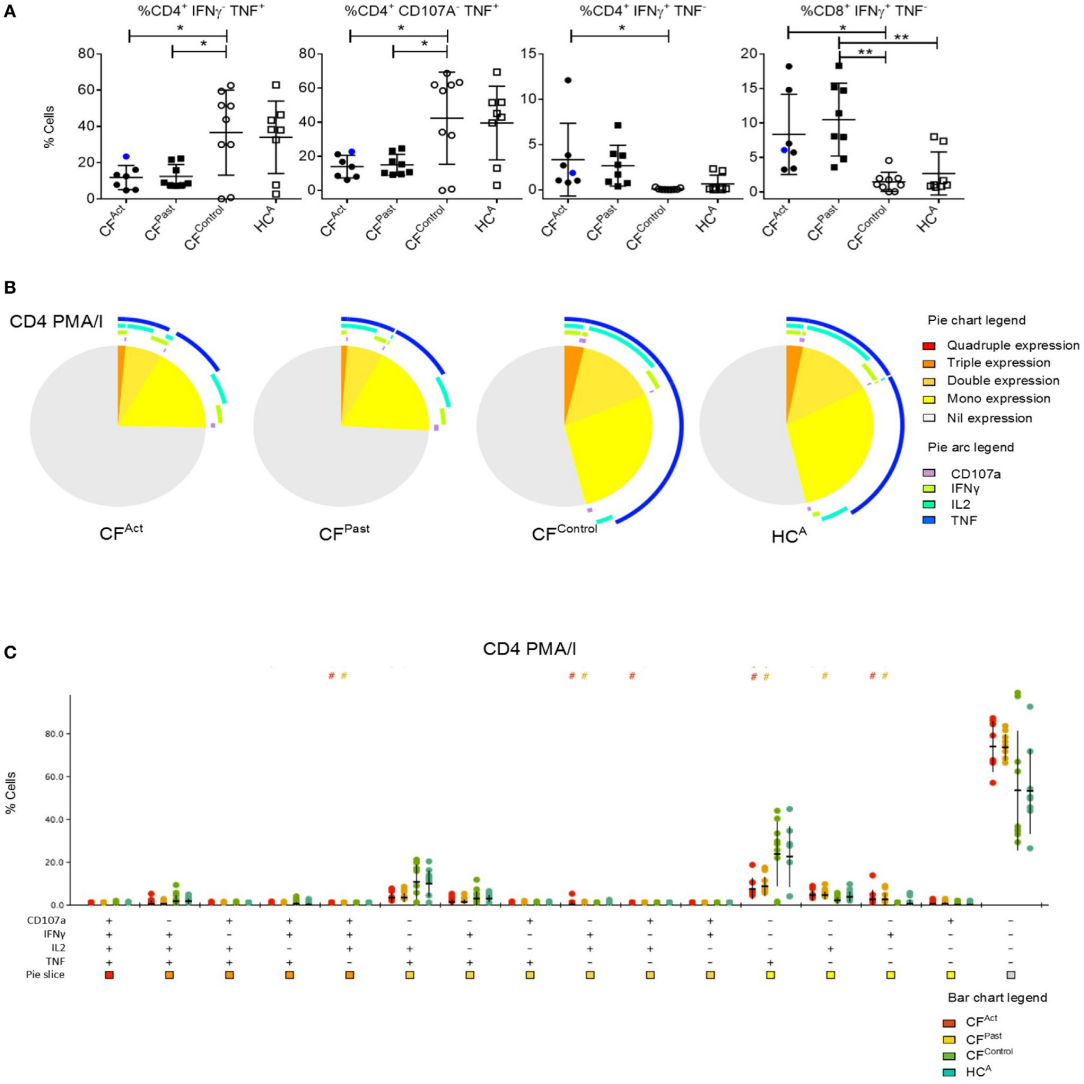
Divergent T cell cytokine profiles in cystic fibrosis (CF) patients based on non-tuberculous mycobacteria (NTM) infection status. **(A)** Flow cytometric analysis of *ex vivo* activated CD4^+^ T cells show significantly lower IFNγ^−^ TNFα^+^ (*P* = 0.026 and *P* = 0.030) and CD107a^−^ TNFα^+^ (*P* = 0.026 and *P* = 0.027) T cells in CF^Act^ and CF^Past^ groups, respectively compared to the CF^Contro1^ group. Differences were not significant when comparing the HC^A^ group with ANOVA *post-hoc* testing. A similar pattern of increased TNFγ^+^ CD4^+^ T cells was seen in the HC^A^ group. Significantly more IFNγ^+^ TNFα^−^ CD4^+^ T cells were seen in CF^Act^ patients compared to CF^Contro1^ patients (*P* = 0.0315) and significantly more IFNγ^+^ TNFα^−^ CD8^+^ T cells were seen in both CF^Act^ and CF^Past^ groups (*P* = 0.014 and *P* = 0.0047, respectively) compared to CF^Contro1^ group. CF^Past^ had significantly more IFNγ^+^ TNFα^−^ CD8^+^ T cells compared to HC^A^(*P* = 0.005). CF^Act^ patient with active *Mycobacterium avium* complex infection is shown as a blue circle in scatter plots. This data point was not included in the ANOVA analysis but is shown here to demonstrate cytokine profile of a patient with an active NTM infection that is not MABS **(B)** Polyfunctionality profiling of CD4^+^ T cells by SPICE showed differences in *ex vivo* functions in CF^Act^ and CF^Past^ groups compared to CF^Control^ (*P* = 0.056 and *P* = 0.041, respectively) and HC^A^ groups (*P* = 0.022 and *P* = 0.012, respectively). TNFα mono-expressing CD4^+^ T cells (blue arc) were significantly lower in the two NTM patient groups compared to the CF^Contro1^ and HC^A^ groups. **(C)** SPICE dot plots show polyfunctionality profile of all combinations of cytokine expression in CD4^+^ T cells after PMA/I stimulation. Groups with significantly different expression compared to CF^Contro1^ (Wilcoxon rank test *P* < 0.05) are indicated with # symbol. Symbol color indicates significantly different group.

*^a^One patient in CF^Act^ group had active MAC infection. This patient was excluded from ANOVA and biomarker analysis but included in general profiling analysis. Data point is shown as a blue circle in scatter plots for ANOVA analysis in Figures 1A and 2A*.

*^b^One patient in CF^Act^ group had prior history of MAC infection while two subsequently developed MAC infection*.

*^c^In NTM^Act^ elderly patient group, one patient had a history of MAC infection prior to MABS infection while two others developed MAC infection after treatment of current episode of MABS infection*.

*^d^Burkholderia cepecia complex*.

Flow cytometric analysis of PBMCs revealed no significant differences in the percentage of B cells (CD19^+^), total CD4^+^ T cells (CD3^+^CD4^+^), total CD8^+^ T cells (CD3^+^CD8^+^), or in TIM-3 expression levels on cell subsets between the CF patients and healthy controls (data not shown). Comparison of Tregs (CD4^+^ CD25^+^ FOXP3^+^) between cohorts showed higher percentages in CF^Act^ and CF^Past^ groups compared to the CF^Control^ group (Figure 1A). Analysis of the individual expression of activation marker CD25 and exhaustion marker CTLA-4 on CD4^+^ T cells revealed significantly higher expression of CD25 and CTLA-4 in CF^Act^ group compared to the CF^Control^ group (Figure 1A). Higher co-expression of CD25 and CTLA-4 was seen on CD4^+^ T cells in CF^Act^ group compared to the CF^Control^ group and higher percentages of CD25 and CTLA-4 double-negative CD4^+^ T cells were observed in the CF^Control^ group and HC^A^ group (Figure 1A).

We next compared the overall pattern of immune marker expression of the CD4^+^ and CD8^+^ T cell compartments in terms of surface “phenotypic fingerprint.” Analysis of triple, double, single, or nil expression of markers CD25, CTLA-4, and PD-1 on CD4^+^ T cells revealed a common fingerprint in CF^Act^ and CF^Past^ groups which was distinct from the CF^Control^ group (Figures 1B,C). A higher number of CLTA-4 single-positive cells were seen in patients with either active or past NTM infection as compared with the healthy control subjects (Figures 1B,C). The T cell fingerprint following PMA/I activation was not significantly different to *ex vivo* T cells (Figures 1B,C). The T cell fingerprint on CD8^+^ T cells was similar between different groups (Figure S1A in Supplementary Material) with significance found in CD8^+^ CD25 single positive T cells *ex vivo* in the CF^Act^ group and in the PMA/I-activated CF^Past^ group (Figure S1A in Supplementary Material). These data reveal a difference in systemic T cell phenotypes in CF patients with active or past NTM disease, particularly in CD4^+^ T cells, compared to CF patients with more common chronic Pa infection. There was no difference between CF patients with active or past NTM disease and healthy controls in terms of T cell fingerprint.

### Distinct T Cell Function in CF Patients Susceptible to MABS Infection

Given the differences in surface T cell phenotypes between cohorts we next analyzed cytokine production post-mitogen stimulation. T cell cytokine production after PMA/I stimulation revealed a specific signature associated with NTM disease. TNFα-producing CD4^+^ T cells were significantly lower in both CF^Act^ and CF^Past^ groups compared to the CF^Control^ group (Figure 2A). TNFα production in CD4^+^ T cells was also markedly lower in both CF^Act^ and CF^Past^ compared to HC^A^ group, though this difference did not reach statistical significance (Bonferroni *post-hoc* test). IFNγ^+^ CD4^+^ T cells in the CF^Act^ group were higher than the CF^Control^ group (Figure 2A). In the CD8^+^ subset, IFNγ^+^ T cells from the CF^Act^ and CF^Past^ groups were higher than the CF^Control^ group (Figure 2A).

Polyfunctionality in CD4^+^ and CD8^+^ T cells was next examined. As CD107a represents degranulation and cytolytic activity, the expression of this marker was included in the polyfunctionality profile in addition to TNFα, IFNγ, and IL-2. Both CF^Act^ and CF^Past^ groups showed a unique polyfunctionality profile compared to both CF^Controls^ and HC^A^ groups (Figure 2B). Both CD4^+^ TNFα-producing single positive T cells (mono-functional) and CD4^+^ TNFα^+^ IL2^+^ double-positive cells (dual-functional) were seen to be significantly reduced in both CF^Act^ and CF^Past^ groups compared to both control groups (Figures 2B,C). Significantly higher IFNγ mono-functional T cells were seen in the CF^Past^ group compared to the CF^Control^ group though there was no difference compared to the HC^A^ group. There was also no difference in the number of triple- and quadruple-functional T cells between groups. When CD8^+^ T cell polyfunctionality was compared, significantly higher numbers of IFNγ producing mono-functional cells were seen in both CF^Act^ and CF^Past^ groups compared to both control groups though there was no significant difference in terms of overall polyfunctionality profile (Figure S2 in Supplementary Material). Total TNFα-producing CD8^+^ T cells were significantly higher in the HC^A^ and CF^Control^ groups compared to CF^Act^ and CF^Past^ groups.

**Figure 3:**
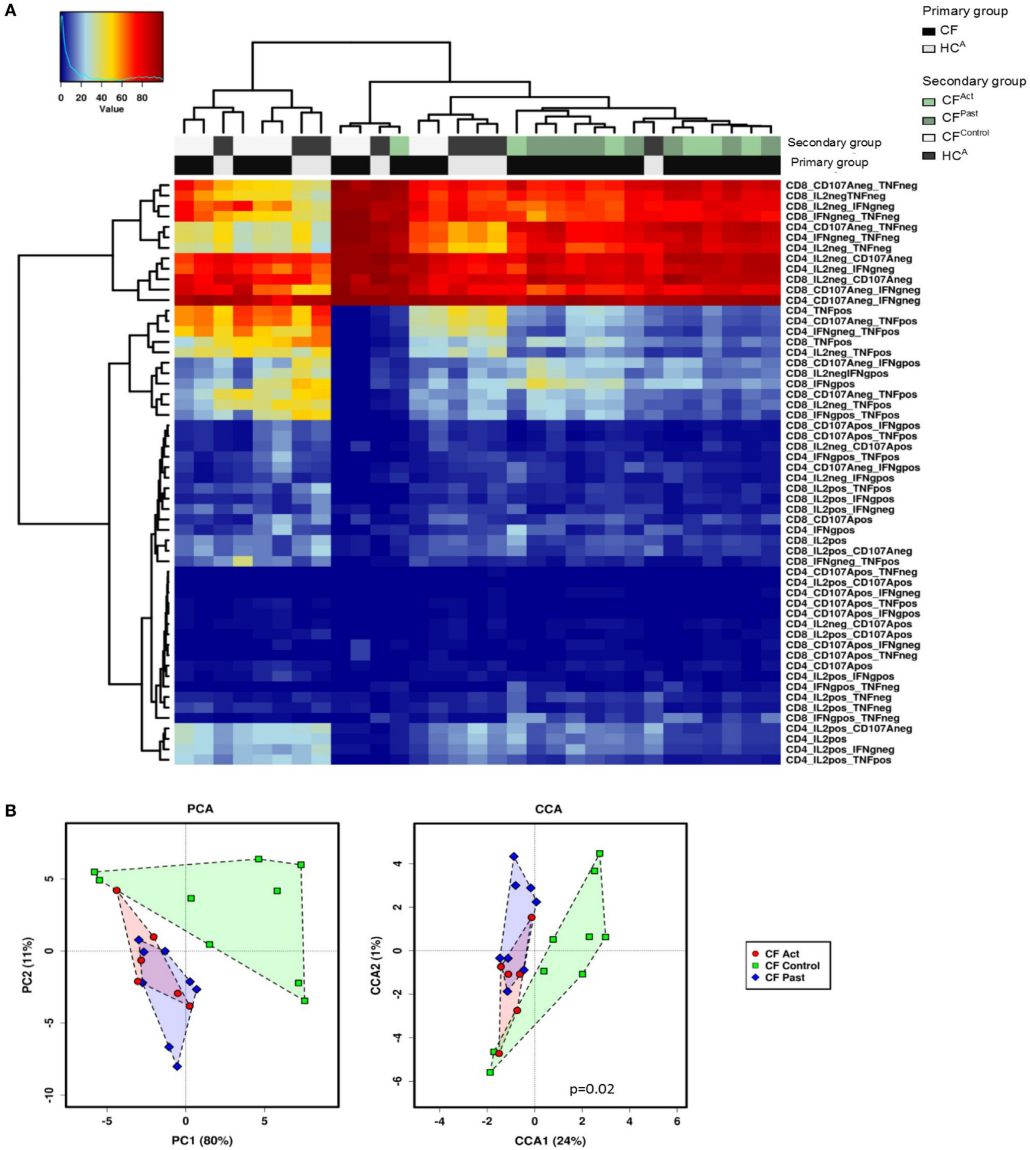
Multivariate T cell analysis of mitogen-stimulated cytokine secretion profiles. **(A)** Unsupervised hierarchical clustering of mitogen-stimulated CD4^+^ and CD8^+^ T cell cytokine secretion profile show divergence based on non-tuberculous mycobacteria infection status. Each column represents an individual (patient/control) and each row represents a clustering variable (cytokine secretion pattern). Clustering patterns shown according to primary group [patient with cystic fibrosis (CF) black, or healthy control gray] and secondary group (CF^Act^-light green, CF^Past^-dark green, CF^Contro1^-white, and HC^A^-black). Clustering of CF^Ac^ and CF^Past^ patients with one exception and clustering of CF^Contro1^ and HC^A^ with one exception. **(B)** Principle component analysis (PCA) and canonical co-variate analysis (CCA) show significant clustering (*P* = 0.02) of subjects by patient group. Marked overlap between CF^Act^ and CF^Past^ groups is seen in terms of global cytokine secretion profile indicating an overall similarity in secretion profile. CF^Contro1^ group diverges further from CF^Act^ and CF^Past^ groups.

Hierarchical clustering analysis of cytokine production and CD107a expression data showed a grouping of CF^Act^ and CF^Past^ groups, while the CF^Control^ and HC^A^ groups clustered together (Figure 3A). Based on global cytokine and CD107a expression profiles, patients with NTM disease (either past or present) could be grouped together. There was no clear separation of the active and past NTM infection groups indicating that based on all clustering variables; no global differences were seen between these two groups. The same pattern was observed in the chronic Pa infection group CF^Control^ and the HC^A^ group where both groups clustered together.

**Figure 4:**
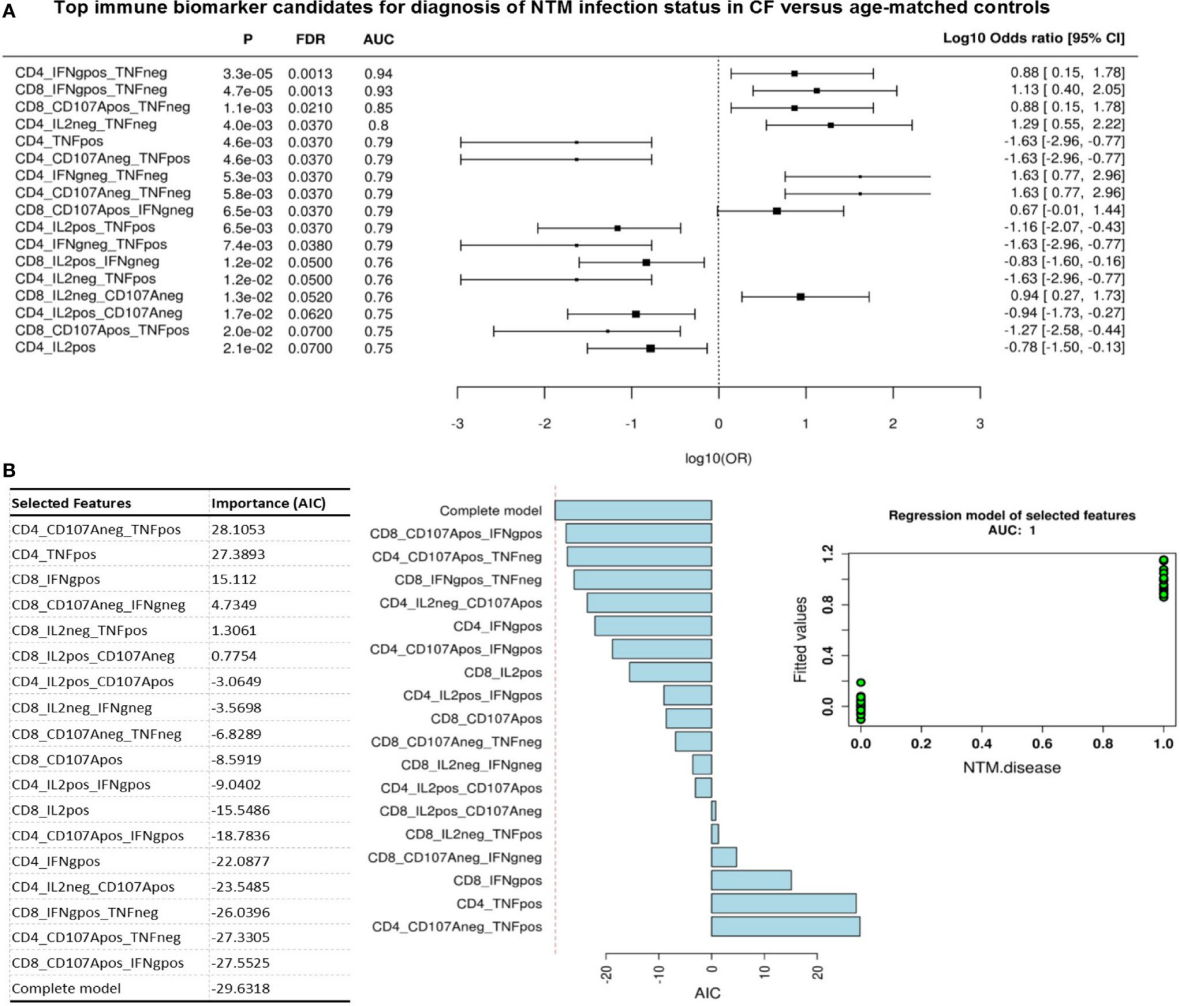
Top immune biomarker candidates for diagnosis of non-tuberculous mycobacteria (NTM) infection status in cystic fibrosis cohorts. **(A)** Flow cytometric biomarkers for NTM disease state from cytokine secretion profile data (Wilcoxon rank test). Graph shows log odds ratio for each biomarker candidate for diagnosis of NTM disease state (negative versus exposed state) with 95% CI. (P, probability; FDR, false discover rate; AUC, area under the curve). **(B)** Stepwise regression model (forward selection) for NTM infection status. Fitted model contains 18 variables shown in table with a final AIC of −30 and an AUC 1. Plots show contribution of each variable to model and fitted value for each patient/control (green dots) when model is applied showing an AUC = 1.

Given this common hierarchical clustering result between CF^Act^ and CF^Past^ cohorts, we next redefined the cohorts for subsequent data analysis. Patients who had either active or past NTM infection (CF^Act^ and CF^Past^) were defined as the “NTM disease” cohort and CF patients with chronic Pa infection and healthy controls (CF^Control^ and HC^A^) were defined as the “control” cohort (i.e., persons with no history of NTM infection). These two variables were then used as outcomes to analyze data for predictive biomarkers using GMine multivariate analysis software (33). CCA analysis showed significant clustering between these groups (Figure 3B). Biomarker analysis identified 13 significant predictors of “NTM disease” after correction for multiple comparisons (FDR) (Figure 4A). Unsurprisingly, significant predictors included combinations of TNFα and IFNγ production. The stepwise regression model (area under the curve, AUC 100%) identified percentage CD8^+^ IL2^+^ TNFα^−^ T cells and percentage CD8^+^ IFNγ^+^ TNFα^−^ T cells as the best predictors of NTM disease (Figure 4B).

### Distinct T Cell Function in Elderly Patients With Active MABS Infection

**Figure 5:**
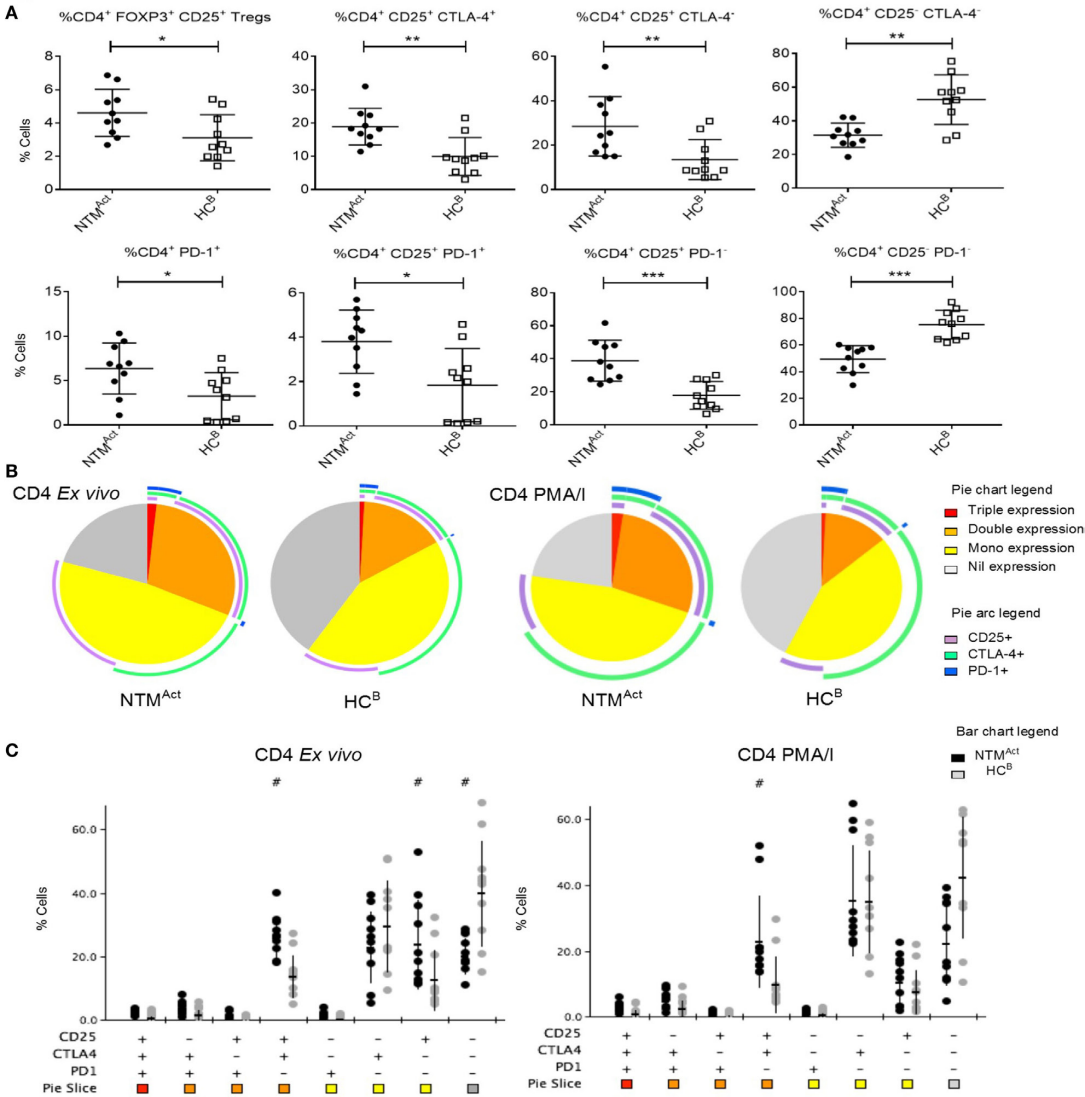
Evidence of CD4^+^ T cell activation and exhaustion in immunocompetent individuals with active non-tuberculous mycobacteria (NTM) infection. **(A)** Flow cytometric analysis of *ex vivo* CD4^+^ T cells showed significantly more Tregs in NTM^Act^ group compared to HC^B^ group (*P* = 0.028). Significantly more CD25^+^ CTLA4^+^ and CD25^+^ CTLA4^−^ CD4^+^ T cells were seen in NTM^Act^ group (*P* = 0.002 and *P* = 0.009, respectively) compared to HC^B^. A reciprocal increase in CD25^−^ CTLA4^−^ CD4^+^ T cells was seen in the HC^B^ group (*P* = 0.001) compared to disease group. Higher numbers of PD1^+^ CD4^+^ T cells (*P* = 0.021) and CD25^+^ PD1^+^ CD4^+^ T cells (*P* = 0.011) were seen in the NTM^Act^ group. CD25^+^ PD1^−^ T cells were significantly higher in NTM^Act^ group (*P* < 0.001) and CD25^−^ and PD1^−^ CD4^+^ T cells were significantly higher in HC^B^ group (*P* < 0.001). **(B)** Phenotyping and polyfunctionality profiling of CD4^+^ T cells by SPICE showed differences in NTM^Act^ and HC^B^ groups directly *ex vivo* and post PMA/I stimulation. Phenotype profiles were significantly different between groups both *ex vivo* (*P* = 0.0013) and post stimulation (*P* = 0.022). Significantly more CD25^+^ CTLA4^+^ T cells were observed in NTM^Act^ groups compared to HC^B^. **(C)** SPICE dot plots show phenotype profile of all combinations of markers CD25, CTLA4, and PD1 in CD4^+^ T cells both *ex vivo* and post PMA/I stimulation between NTM^Act^ and HC^B^ groups. Significantly different expression compared to HC^B^ (Wilcoxon rank test *P* < 0.05) is indicated with # symbol.

To determine whether this immune profile would also be found in other independent disease cohorts, we next investigated elderly patients with active NTM infection (NTM^Act^). In Australia, the rate of notified NTM cases per 100,000 population has increased by approximately 17% per year between 2012 and 2015 (35). The reasons for this increase are unknown, and there is yet no predictor to identify at-risk individuals. To determine if underlying immune dysfunction may be a predictive factor for NTM infection we compared NTM^Act^ patients with elderly healthy controls (HC^B^). We found that Tregs were increased in the peripheral blood of elderly NTM^Act^ patients compared to elderly HC^B^ (Figure 5A). Elevated CD25 and CTLA-4 expression was also seen on CD4^+^ T cells in the NTM^Act^ group alongside increased PD-1 expression on CD4^+^ CD25^+^ T cells. Analysis of the phenotypic fingerprint of CD25, CTLA-4, and PD-1 expression in CD4^+^ T cells showed a specific signature in NTM^Act^ patients with elevated CD25 and CTLA-4 double-positive T cells as well as elevated CD25 single-positive T cells in the NTM^Act^ group (Figures 5A,B). T cells negative for all three markers (triple-negative) were significantly higher in the HC^B^ group compared to NTM^Act^ patients. Four significant phenotypic differences *ex vivo* and during mitogen stimulation were observed between the NTM^Act^ group and HC^B^ group (Figure 5C). *Ex vivo* CD8^+^ T cell fingerprint was identical between HC^B^ and NTM^Act^ cohorts (data not shown).

**Figure 6:**
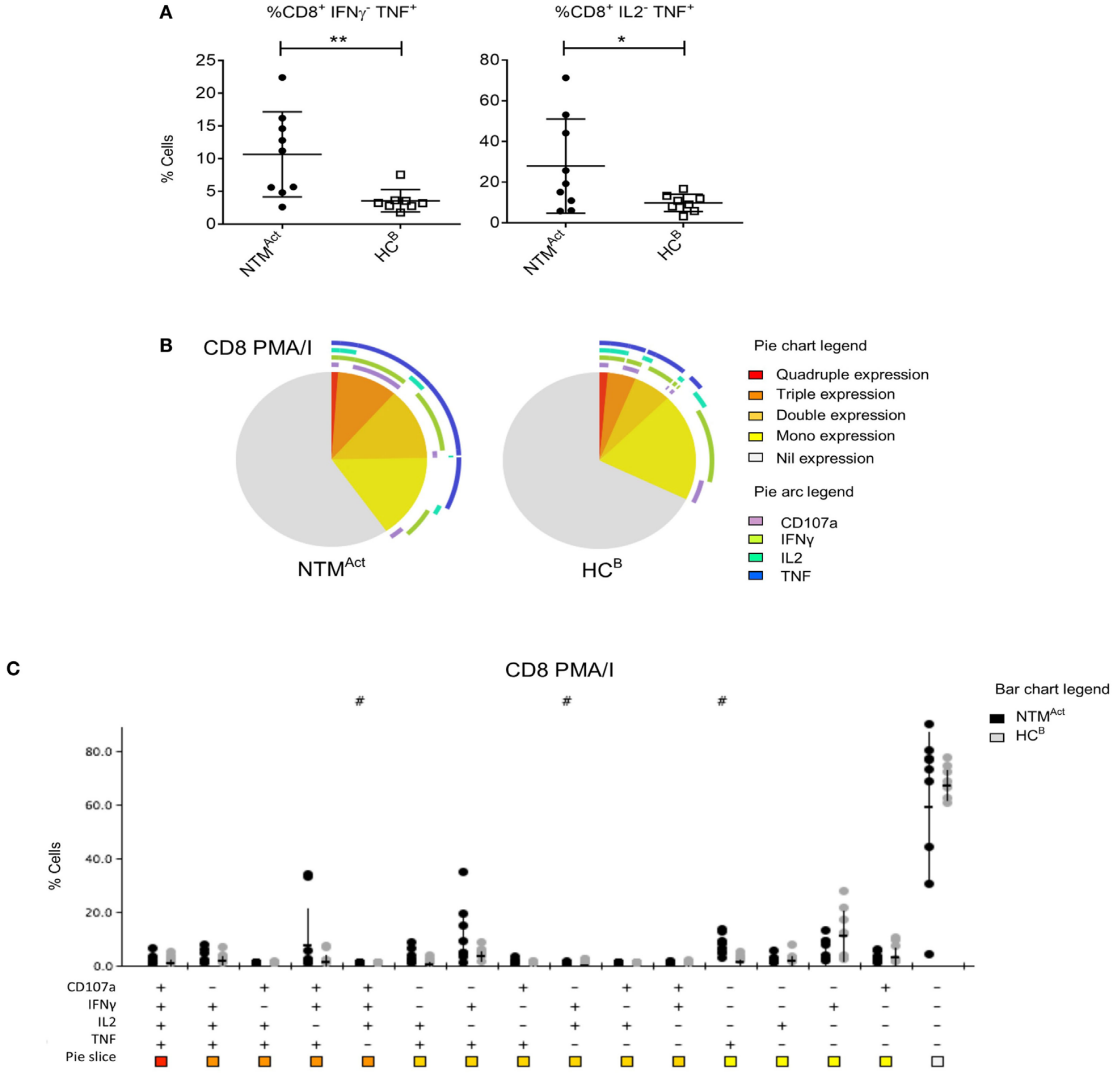
Divergence of CD8^+^ T cell cytokine profiles in immunocompetent individuals with active non-tuberculous mycobacteria (NTM) infection. **(A)** Flow cytometric analysis of *ex vivo* CD8^+^ T cells showed significantly more IFNγ^−^ TNFα^+^ cells (*P* = 0.01) and IL2^−^ TNFα^+^ cells (*P* = 0.048) in NTM^Act^ compared to HC^B^. **(B)** Polyfunctionality profiling of CD8^+^ T cells shown in pie charts were not significantly different between the two groups. However, significantly higher TNFα mono-functional CD8^+^ T cells were observed in NTM patient group (*P* = 0.002). **(C)** SPICE dot plots show polyfunctionality profile of all combinations of cytokine expression in CD8^+^ T cells after PMA/I stimulation between NTM^Act^ and HC^B^ groups. Significantly different expression compared to HC^B^ (Wilcoxon rank test *P* < 0.05) is indicated with # symbol.

Mitogen stimulation and polyfunctionality analysis in the elderly cohorts revealed a very different profile to that seen in CF patients. In the NTM^Act^ group, significantly higher TNFα production by CD8^+^ T cells was observed (Figure 6A), while there was no difference in the number of TNFα-producing CD4^+^ T cells between groups (Figure S3A in Supplementary Material). IFNγ single-positive CD8^+^ T cells were similar in patients and controls (Figures 6B,C) as was the overall polyfunctionality profile in CD4^+^ T cells (Figures S3A,B in Supplementary Material).

## Discussion

The increased incidence and prevalence of NTM disease in recent years, warrants a more comprehensive understanding of immunity in susceptible individuals (7). Due to increasing antibiotic resistance of NTM strains and poor patient-outcomes in particular MABS, immunomodulatory strategies may emerge as important adjuvants to conventional anti-mycobacterial therapy in NTM disease. Here, we dissect faults in immunity in two cohorts of patients (CF and elderly) with NTM lung infection and matched controls to identify the underlying blood immune signatures of each disease. Blood was examined given: (i) T cells are known to traffic between the blood and lung (36); (ii) NTM-specific T cells have been observed in the blood (37); and (iii) blood is easy to access for diagnostic tests. The first cohort included CF patients with active NTM disease, CF patients with past NTM disease who had been successfully treated and were now in disease remission, CF patients with chronic Pa infection who had no history of NTM infection and a group of matched healthy controls. The second cohort included elderly patients with NTM infection and matched healthy controls. All control individuals in both cohorts had active or past MABS infection with the exception of one patient who had MAC infection.

The frequency of Tregs was significantly increased in CF patients with active and past NTM infection compared to CF patients with chronic Pa infection. These data contrast with the elderly cohort, where Tregs significantly were higher in elderly NTM patients compared to healthy controls. This was similarly observed by Hector et al., where lower number of Tregs was found in both the airways and peripheral blood of CF patients compared to healthy controls and a further reduction in Tregs was seen in patients with chronic Pa infection (38). The lack of difference in Treg percentages between CF NTM patients and healthy controls could be an indication of an increase in Tregs following NTM infection. This rationale would align with our findings of elevated Tregs in the elderly immunocompetent patients with active NTM infection. Of note, increased Treg numbers have been observed in the peripheral blood of TB patients (39–41) indicating overlap between TB and NTM immunopathology.

When examining the T cell fingerprint, CF patients with active and past MABS infection were different to control patients with chronic Pa infection, though they were similar to the healthy control group. This finding suggests differences in T cell immunity between CF patients susceptible to MABS and CF patients who have no history of NTM infection.

Distinct activation and exhaustion profiles were seen in both patient cohorts with NTM infection compared to corresponding controls. Higher numbers of CD4^+^ T cells co-expressing the activation marker CD25 and the exhaustion marker CTLA-4 were seen in CF patients with both active and past NTM infection. In the elderly cohort, a similar pattern of increased CD25 and CTLA-4 co-expression was observed on CD4^+^ T cells. PD-1 expression was also increased on CD4^+^ T cells, suggesting an exhausted immune phenotype. High PD-1 expression on T cells associates with increased TB disease burden (26). Increased PD-1 expression on T cells, B cells, NK cells, and monocytes has also been reported in patients with MAC infection (42). In mice, PD-1 gene knockout can enhance TB resistance by preventing over-production of IFNγ (43). However, another study showed that PD-1 gene knockout mice can be more susceptible to NTM infection (44). To date, the immune checkpoint CTLA-4 has not been studied in the context of NTM. Our data present the first finding of elevated CTLA-4 on T cells in NTM infection. The significance of this as well as elevated PD-1 expression on T cells indicates a degree of immune suppression in NTM infection. No PD-1 (or CTLA-4) antibody blockade therapy has been examined *in vivo* on TB or NTM. Future research should focus on the prospect of treating NTM patients using PD-1 and/or CTLA-4 antibody blockade.

TNFα directly activates macrophages to restrict mycobacterial growth and induces apoptosis of infected macrophages leading to bacterial killing (45–47). TNFα is also essential for granuloma formation and disease restriction during mycobacterial pathogenesis highlighting its importance for *in vivo* control of the pathogen (48). Animals deficient in TNFα are highly susceptible to disseminated forms of TB (49). The cytokine polyfunctionality profiles seen in CF cohorts revealed underlying global TNFα deficiencies that could explain susceptibility to NTM infection in CF patients. Low TNFα secretion (mono-functional TNFα-secreting CD4^+^ T cells) was seen in CF patients with both active and past NTM infection, while increased TNFα secreting mono-functional CD8^+^ T cells were seen in elderly patients with NTM infection. Given that CF patients with both active and past infection exhibit this TNFα deficiency in response to mitogen, it is more likely that this phenotype is an underlying predisposition to disease rather than a direct effect of the disease. The contrasting pattern of TNFα secretion in the two disease scenarios is significant in the larger context of mycobacterial pathobiology. Studies of NTM immunity are conflicting and show both low and high levels of TNFα production (12, 42, 50–52), however, these studies did not perform in depth mapping of cell subsets. TNFα production has an important role in host resistance as treatment with anti-TNFα therapies are associated with increased susceptibility to active TB and reactivation of latent TB infection (53) and is correlated with NTM activation in autoimmune diseases, such as rheumatoid arthritis, Crohn’s disease, ankylosing spondylitis, and psoriasis (54). Here, we have shown that the TNFα profiles can vary with disease setting. Thus, taking into account the individual patient profile is critical when interpreting these findings. Moreover, we have also shown that TNFα secretion levels vary according to cell subset and it may be specific deficiencies in certain cell subsets that predispose to disease. It is possible that low dose TNFα replacement therapy, *via* aerosol, for example, may aid standard of care in NTM treatment in CF. Indeed, other cytokine replacement therapies, such as IFNγ, IFNα, IL-2, GM-CSF, and IL-12 have shown promise against TB, MDR-TB, MAC, and MABS [reviewed in Ref. (53)]. Systemically administered IFNγ has shown the most promise for clinical use (55).

The importance of IFNγ in anti-mycobacterial immunity is widely accepted and extensively studied in TB (56). Reduced IFNγ production in NTM infection has been shown in several studies (51, 57, 58) though contradictory results have also been reported (16). Akin to TNFα, we show that IFNγ secretion varies with cell subset and disease scenario. In the CF NTM cohort, increased IFNγ secretion was seen in both CD4^+^ and CD8^+^ T cells compared to healthy controls, whereas in the elderly NTM cohort, there was no significant increase in IFNγ-secreting cells in both CD4^+^ or CD8^+^ T cells. This could indicate an inadequate protective response rather than a deficiency of cytokine. Comparison of MABS infection in these two disease cohorts once again highlights the importance of the clinical context when searching for risk factors. However, one limitation of the study is the profiling of circulating lymphocytes which may differ to those at the site of disease.

The search for environmental and behavioral risk factors for NTM infection in CF patients has found increased acquisition in the tropics and decreased acquisition with macrolide treatment (59). Here, we show that it is also possible to define immune parameters in the circulatory blood that help to identify at-risk individuals. Specifically, we showed that CF patients can stratify CF into two groups based on Treg frequencies, CD4^+^ T cell surface phenotype (CD25 and CTLA-4) and cytokine production by CD4^+^/CD8^+^ T cells (IFNγ, TNFα, CD107a, and IL-2), with at-risk patients exhibiting a distinct deficiency in TNFα. We suggest that this immune signature could be further refined and validated in independent cohort studies, and then ultimately developed for use as a diagnostic tool to identify individuals who are at high risk of developing NTM infection. Targeted behavioral interventions for at-risk patients may subsequently decrease the risk of NTM acquisition from the environment or infected patients.

The role of CD8^+^ T cells in NTM infection in humans remains unclear. Research in TB in primate and mouse models has shown that CD8^+^ T cells are important in controlling experimental infection. However, the precise mechanism by which they contribute to protection is unknown (60). Regulatory CD8^+^ T cells have been identified in human TB, though their role in protection is also not clear (60).

This study specifically focused on MABS infection. It is important to highlight that the CF patient with active MAC infection (shown as a blue circle in Figures 1A and 2A) exhibited a T cell fingerprint and T cell functional profile very similar to patients with MABS infection (note; this patient was not included in statistical analysis). Additionally, it is also important to note the 2 of 6 CF MABS patients and 3 of 10 elderly MABS patients either had a previous history of MAC infection or subsequently developed MAC infection. This tentatively suggests that the susceptibility to NTM infection may occur through a common immune dysfunction pathway, although further antigen-specific immune studies are required to validate this hypothesis. If NTM-specific immune dysfunction is common in the individuals, targeted immunotherapies may help in correcting this deficit.

Limitations of this study include the small sample size due to restrictions in patient recruitment within a confined study duration. An additional, larger patient cohort will be essential to validate our predictive model. Limitations in sample volumes also restricted the analysis to *ex vivo* phenotyping and mitogen-activation signatures. Thus, antigen-specific immune responses were not conducted in this study. However, further research is warranted given the identification of significant global immune anomalies in this exploratory study. Anomalies in mitogen-triggered activation signatures in the global T cell compartment suggest an underlying immune deficiency in the patients that would likely translate to NTM-specific responses as seen in TB (61, 62).

In summary, our study presents the first data on immune checkpoint expression on T cell subsets in human MABS infection as well as the first comparison of T cell polyfuntionality between CF and non-CF patient groups with MABS infection in response to mitogen stimulation. We show that MABS infection in two different patient groups’ exhibit specific immune phenotypes and show dysregulation in type 1 cytokine production and a global decrease in T cell “quality.” In CF patients, TNFα-mediated immunity may hold the key to understanding the increased risk of MABS infection and guide future therapeutic interventions. In elderly individuals, interference with checkpoint molecules (PD-1 and CTLA-4) may guide future therapeutic interventions. Collectively, the study has revealed many potential associations between T cell phenotype and individuals at risk of MABS infection. The idea of an underlying immune dysfunction which predisposes certain individuals to NTM infection is attractive but very speculative. Larger populations and further functional experiments will be required to validate this hypothesis.

## Ethics Statement

All samples were obtained with written consent and all protocols were approved by the Human Research Ethics Committees of the QIMR Berghofer Medical Research Institute, The Prince Charles Hospital, and Greenslopes Private Hospital, Australia (QIMR Berghofer HREC P2045).

## Author Contributions

SB and JM conceptualized the study. VL, CR, DS, AK, DD, DR, RT, and JM performed the experiments and analyzed the data. CR, VL, and JM wrote the manuscript with input from all authors. DR, RT, SB, and JM supervised the study. All authors approved the final manuscript.

## Conflict of Interest Statement

The authors declare that the research was conducted in the absence of any commercial or financial relationships that could be construed as a potential conflict of interest.

## Footnotes

^1^www.Cytobank.org.

^2^http://cgenome.net/GMine/.

^3^https://exon.niaid.nih.gov/spice/.

## References

[1] Morimoto K Iwai K Uchimura K Okumura M Yoshiyama T Yoshimori K A steady increase in nontuberculous mycobacteriosis mortality and estimated prevalence in Japan. Ann Am Thorac Soc (2014) 11(1):1–8. 10.1513/AnnalsATS.201303-067OC 24102151

[2] Society TRCotBT. Pulmonary disease caused by *Mycobacterium avium*-intracellulare in HIV-negative patients: five-year follow-up of patients receiving standardised treatment. Int J Tuberc Lung Dis (2002) 6(7):628–34. 12102303

[3] Andrejak C Thomsen VO Johansen IS Riis A Benfield TL Duhaut P Nontuberculous pulmonary mycobacteriosis in Denmark: incidence and prognostic factors. Am J Respir Crit Care Med (2010) 181(5):514–21. 10.1164/rccm.200905-0778OC 20007929

[4] Fleshner M Olivier KN Shaw PA Adjemian J Strollo S Claypool RJ Mortality among patients with pulmonary non-tuberculous mycobacteria disease. Int J Tuberc Lung Dis (2016) 20(5):582–7. 10.5588/ijtld.15.0807 27084809 PMC6660916

[5] Fangous MS Mougari F Gouriou S Calvez E Raskine L Cambau E Classification algorithm for subspecies identification within the *Mycobacterium abscessus* species, based on matrix-assisted laser desorption ionization-time of flight mass spectrometry. J Clin Microbiol (2014) 52(9):3362–9. 10.1128/JCM.00788-14 25009048 PMC4313163

[6] Hoefsloot W van Ingen J Andrejak C Angeby K Bauriaud R Bemer P The geographic diversity of nontuberculous mycobacteria isolated from pulmonary samples: an NTM-NET collaborative study. Eur Respir J (2013) 42(6):1604–13. 10.1183/09031936.00149212 23598956

[7] Prevots DR Marras TK. Epidemiology of human pulmonary infection with nontuberculous mycobacteria: a review. Clin Chest Med (2015) 36(1):13–34. 10.1016/j.ccm.2014.10.002 25676516 PMC4332564

[8] Henkle E Aksamit T Barker A Daley CL Griffith D Leitman P Patient-centered research priorities for pulmonary nontuberculous mycobacteria (NTM) infection. An NTM Research Consortium Workshop Report. Ann Am Thorac Soc (2016) 13(9):S379–84. 10.1513/AnnalsATS.201605-387WS 27627485 PMC5461946

[9] Bryant JM Grogono DM Rodriguez-Rincon D Everall I Brown KP Moreno P Emergence and spread of a human-transmissible multidrug-resistant nontuberculous mycobacterium. Science (2016) 354(6313):751–7. 10.1126/science.aaf8156 27846606 PMC5142603

[10] Martiniano SL Nick JA Daley CL. Nontuberculous mycobacterial infections in cystic fibrosis. Clin Chest Med (2016) 37(1):83–96. 10.1016/j.ccm.2015.11.001 26857770

[11] Qvist T Gilljam M Jonsson B Taylor-Robinson D Jensen-Fangel S Wang M Epidemiology of nontuberculous mycobacteria among patients with cystic fibrosis in Scandinavia. J Cyst Fibros (2015) 14(1):46–52. 10.1016/j.jcf.2014.08.002 25178871 PMC4298356

[12] Bar-On O Mussaffi H Mei-Zahav M Prais D Steuer G Stafler P Increasing nontuberculous mycobacteria infection in cystic fibrosis. J Cyst Fibros (2015) 14(1):53–62. 10.1016/j.jcf.2014.05.008 24917112

[13] Park IK Olivier KN. Nontuberculous mycobacteria in cystic fibrosis and non-cystic fibrosis bronchiectasis. Semin Respir Crit Care Med (2015) 36(2):217–24. 10.1055/s-0035-1546751 25826589 PMC7171444

[14] Chan ED Iseman MD. Underlying host risk factors for nontuberculous mycobacterial lung disease. Semin Respir Crit Care Med (2013) 34(1):110–23. 10.1055/s-0033-1333573 23460011

[15] Chan ED Bai X Kartalija M Orme IM Ordway DJ. Host immune response to rapidly growing mycobacteria, an emerging cause of chronic lung disease. Am J Respir Cell Mol Biol (2010) 43(4):387–93. 10.1165/rcmb.2009-0276TR 20081053

[16] Lim A Allison C Price P Waterer G. Susceptibility to pulmonary disease due to *Mycobacterium avium*-intracellulare complex may reflect low IL-17 and high IL-10 responses rather than Th1 deficiency. Clin Immunol (2010) 137(2):296–302. 10.1016/j.clim.2010.07.011 20797909

[17] Cooper AM Mayer-Barber KD Sher A. Role of innate cytokines in mycobacterial infection. Mucosal Immunol (2011) 4(3):252–60. 10.1038/mi.2011.13 21430655 PMC3294290

[18] Kim SY Koh WJ Kim YH Jeong BH Park HY Jeon K Importance of reciprocal balance of T cell immunity in *Mycobacterium abscessus* complex lung disease. PLoS One (2014) 9(10):e109941. 10.1371/journal.pone.0109941 25295870 PMC4190320

[19] Matsuyama M Ishii Y Yageta Y Ohtsuka S Ano S Matsuno Y Role of Th1/Th17 balance regulated by T-bet in a mouse model of *Mycobacterium avium* complex disease. J Immunol (2014) 192(4):1707–17. 10.4049/jimmunol.1302258 24446514

[20] Becker KL van Ingen J Ten Oever J Merkus PJ Ferwerda G Netea MG Deficient interleukin-17 production in response to *Mycobacterium abscessus* in cystic fibrosis. Eur Respir J (2016) 47(3):990–3. 10.1183/13993003.00446-2015 26743483

[21] Allie N Alexopoulou L Quesniaux VJ Fick L Kranidioti K Kollias G Protective role of membrane tumour necrosis factor in the host’s resistance to mycobacterial infection. Immunology (2008) 125(4):522–34. 10.1111/j.1365-2567.2008.02865.x 18544042 PMC2612548

[22] Hoos A. Development of immuno-oncology drugs – from CTLA4 to PD1 to the next generations. Nat Rev Drug Discov (2016) 15(4):235–47. 10.1038/nrd.2015.35 26965203

[23] Kaufmann DE Walker BD. PD-1 and CTLA-4 inhibitory cosignaling pathways in HIV infection and the potential for therapeutic intervention. J Immunol (2009) 182(10):5891–7. 10.4049/jimmunol.0803771 19414738 PMC3726306

[24] Smith P Walsh CM Mangan NE Fallon RE Sayers JR McKenzie AN *Schistosoma mansoni* worms induce anergy of T cells via selective up-regulation of programmed death ligand 1 on macrophages. J Immunol (2004) 173(2):1240–8. 10.4049/jimmunol.173.2.1240 15240716

[25] Das S Suarez G Beswick EJ Sierra JC Graham DY Reyes VE. Expression of B7-H1 on gastric epithelial cells: its potential role in regulating T cells during *Helicobacter pylori* infection. J Immunol (2006) 176(5):3000–9. 10.4049/jimmunol.176.5.3000 16493058

[26] Jurado JO Alvarez IB Pasquinelli V Martinez GJ Quiroga MF Abbate E Programmed death (PD)-1:PD-ligand 1/PD-ligand 2 pathway inhibits T cell effector functions during human tuberculosis. J Immunol (2008) 181(1):116–25. 10.4049/jimmunol.181.1.116 18566376

[27] McNab FW Berry MP Graham CM Bloch SA Oni T Wilkinson KA Programmed death ligand 1 is over-expressed by neutrophils in the blood of patients with active tuberculosis. Eur J Immunol (2011) 41(7):1941–7. 10.1002/eji.201141421 21509782 PMC3179592

[28] Szaba FM Kummer LW Duso DK Koroleva EP Tumanov AV Cooper AM TNFalpha and IFNgamma but not perforin are critical for CD8 T cell-mediated protection against pulmonary Yersinia pestis infection. PLoS Pathog (2014) 10(5):e1004142 10.1371/journal.ppat.1004142 24854422 PMC4031182

[29] Frey O Meisel J Hutloff A Bonhagen K Bruns L Kroczek RA Inducible costimulator (ICOS) blockade inhibits accumulation of polyfunctional T helper 1/T helper 17 cells and mitigates autoimmune arthritis. Ann Rheum Dis (2010) 69(8):1495–501. 10.1136/ard.2009.119164 20498202

[30] Prezzemolo T Guggino G La Manna MP Di Liberto D Dieli F Caccamo N. Functional signatures of human CD4 and CD8 T cell responses to *Mycobacterium tuberculosis*. Front Immunol (2014) 5:180. 10.3389/fimmu.2014.00180 24795723 PMC4001014

[31] Lee TWR Brownlee KG Conway SP Denton M Littlewood JM Evaluation of a new definition for chronic *Pseudomonas aeruginosa* infection in cystic fibrosis patients. J Cyst Fibros (2003) 2(1):29–34. 10.1016/S1569-1993(02)00141-8 15463843

[32] Amir el AD Davis KL Tadmor MD Simonds EF Levine JH Bendall SC viSNE enables visualization of high dimensional single-cell data and reveals phenotypic heterogeneity of leukemia. Nat Biotechnol (2013) 31(6):545–52. 10.1038/nbt.2594 23685480 PMC4076922

[33] Proietti C Zakrzewski M Watkins TS Berger B Hasan S Ratnatunga CN Mining, visualizing and comparing multidimensional biomolecular data using the Genomics Data Miner (GMine) Web-Server. Sci Rep (2016) 6(1):38178. 10.1038/srep38178 27922118 PMC5138638

[34] Roederer M Nozzi JL Nason MC. SPICE: exploration and analysis of post-cytometric complex multivariate datasets. Cytometry A (2011) 79(2):167–74. 10.1002/cyto.a.21015 21265010 PMC3072288

[35] Thomson R Donnan E Konstantinos A. Notification of nontuberculous mycobacteria: an Australian perspective. Ann Am Thorac Soc (2017) 14(3):318–23. 10.1513/AnnalsATS.201612-994OI 28118021

[36] Jennrich S Lee MH Lynn RC Dewberry K Debes GF. Tissue exit: a novel control point in the accumulation of antigen-specific CD8 T cells in the influenza a virus-infected lung. J Virol (2012) 86(7):3436–45. 10.1128/JVI.07025-11 22278253 PMC3302526

[37] Steindor M Nkwouano V Mayatepek E Mackenzie CR Schramm D Jacobsen M. Rapid detection and immune characterization of *Mycobacterium abscessus* infection in cystic fibrosis patients. PLoS One (2015) 10(3):e0119737. 10.1371/journal.pone.0119737 25742660 PMC4351040

[38] Hector A Schafer H Poschel S Fischer A Fritzsching B Ralhan A Regulatory T-cell impairment in cystic fibrosis patients with chronic pseudomonas infection. Am J Respir Crit Care Med (2015) 191(8):914–23. 10.1164/rccm.201407-1381OC 25632992

[39] Guyot-Revol V Innes JA Hackforth S Hinks T Lalvani A. Regulatory T cells are expanded in blood and disease sites in patients with tuberculosis. Am J Respir Crit Care Med (2006) 173(7):803–10. 10.1164/rccm.200508-1294OC 16339919

[40] Ribeiro-Rodrigues R Resende Co T Rojas R Toossi Z Dietze R Boom WH A role for CD4+CD25+ T cells in regulation of the immune response during human tuberculosis. Clin Exp Immunol (2006) 144(1):25–34. 10.1111/j.1365-2249.2006.03027.x 16542361 PMC1809641

[41] Chen X Zhou B Li M Deng Q Wu X Le X CD4(+)CD25(+)FoxP3(+) regulatory T cells suppress *Mycobacterium tuberculosis* immunity in patients with active disease. Clin Immunol (2007) 123(1):50–9. 10.1016/j.clim.2006.11.009 17234458

[42] Shu CC Wang JY Wu MF Wu CT Lai HC Lee LN Attenuation of lymphocyte immune responses during *Mycobacterium avium* complex-induced lung disease due to increasing expression of programmed death-1 on lymphocytes. Sci Rep (2017) 7:42004. 10.1038/srep42004 28169347 PMC5294633

[43] Sakai S Kauffman KD Sallin MA Sharpe AH Young HA Ganusov VV CD4 T Cell-Derived IFN-gamma plays a minimal role in control of pulmonary *Mycobacterium tuberculosis* infection and must be actively repressed by PD-1 to prevent lethal disease. PLoS Pathog (2016) 12(5):e1005667 10.1371/journal.ppat.1005667 27244558 PMC4887085

[44] Barber DL Mayer-Barber KD Feng CG Sharpe AH Sher A. CD4 T cells promote rather than control tuberculosis in the absence of PD-1-mediated inhibition. J Immunol (2011) 186(3):1598–607. 10.4049/jimmunol.1003304 21172867 PMC4059388

[45] Jayaraman P Sada-Ovalle I Nishimura T Anderson AC Kuchroo VK Remold HG IL-1beta promotes antimicrobial immunity in macrophages by regulating TNFR signaling and caspase-3 activation. J Immunol (2013) 190(8):4196–204. 10.4049/jimmunol.1202688 23487424 PMC3622150

[46] Balcewicz-Sablinska MK Keane J Kornfeld H Remold HG. Pathogenic *Mycobacterium tuberculosis* evades apoptosis of host macrophages by release of TNF-R2, resulting in inactivation of TNF-alpha. J Immunol (1998) 161(5):2636–41. 9725266

[47] Bermudez LE Young LS Tumor necrosis factor, alone or in combination with IL-2, but not IFN-gamma, is associated with macrophage killing of *Mycobacterium avium* complex. J Immunol (1988) 140(9):3006–13. 2834450

[48] Roach DR Bean AGD Demangel C France MP Briscoe H Britton WJ. TNF regulates chemokine induction essential for cell recruitment, granuloma formation, and clearance of mycobacterial infection. J Immunol (2002) 168(9):4620–7. 10.4049/jimmunol.168.9.4620 11971010

[49] Flynn JL Goldstein MM Chan J Triebold KJ Pfeffer K Lowenstein CJ Tumor necrosis factor-alpha is required in the protective immune response against *Mycobacterium tuberculosis* in mice. Immunity (1995) 2(6):561–72. 10.1016/1074-7613(95)90001-2 7540941

[50] Greinert U Schlaak M Rusch-Gerdes S Flad HD Ernst M. Low in vitro production of interferon-gamma and tumor necrosis factor-alpha in HIV-seronegative patients with pulmonary disease caused by nontuberculous mycobacteria. J Clin Immunol (2000) 20(6):445–52. 10.1023/A:1026407815946 11202234

[51] Kwon YS Kim EJ Lee SH Suh GY Chung MP Kim H Decreased cytokine production in patients with nontuberculous mycobacterial lung disease. Lung (2007) 185(6):337–41. 10.1007/s00408-007-9040-z 17926095

[52] Sampaio EP Elloumi HZ Zelazny A Ding L Paulson ML Sher A *Mycobacterium abscessus* and *M. avium* trigger toll-like receptor 2 and distinct cytokine response in human cells. Am J Respir Cell Mol Biol (2008) 39(4):431–9. 10.1165/rcmb.2007-0413OC 18441280 PMC2551704

[53] Tomioka H. Adjunctive immunotherapy of mycobacterial infections. Curr Pharm Des (2004) 10(26):3297–312. 10.2174/1381612043383232 15544517

[54] Desai AA Marks DJ Atypical mycobacteria: showerheads, anti-TNF therapy and Crohn’s disease. Expert Rev Clin Immunol (2010) 6(5):695–9. 10.1586/eci.10.61 20828275

[55] Reljic R Paul MJ Arias MA. Cytokine therapy of tuberculosis at the crossroads. Expert Rev Respir Med (2009) 3(1):53–66. 10.1586/17476348.3.1.53 20477282

[56] Kumar P IFNgamma-producing CD4+ T lymphocytes: the double-edged swords in tuberculosis. Clin Transl Med (2017) 6(1):21 10.1186/s40169-017-0151-8 28646367 PMC5482791

[57] Vankayalapati R Wizel B Samten B Griffith DE Shams H Galland MR Cytokine profiles in immunocompetent persons infected with *Mycobacterium avium* complex. J Infect Dis (2001) 183(3):478–84. 10.1086/318087 11133380

[58] Safdar A White DA Stover D Armstrong D Murray HW Profound interferon gamma deficiency in patients with chronic pulmonary nontuberculous mycobacteriosis. Am J Med (2002) 112:756–9. 10.1016/S0002-9343(02)01313-X 12517367

[59] Sherrard LJ Tay GT Butler CA Wood ME Yerkovich S Ramsay KA Tropical Australia is a potential reservoir of non-tuberculous mycobacteria in cystic fibrosis. Eur Respir J (2017) 49(5):1–4. 10.1183/13993003.00046-2017 28495693

[60] Lin PL Flynn JL. CD8 T cells and *Mycobacterium tuberculosis* infection. Semin Immunopathol (2015) 37(3):239–49. 10.1007/s00281-015-0490-8 25917388 PMC4439333

[61] Riou C Berkowitz N Goliath R Burgers WA Wilkinson RJ. Analysis of the phenotype of *Mycobacterium tuberculosis*-specific CD4+ T cells to discriminate latent from active tuberculosis in HIV-uninfected and HIV-infected individuals. Front Immunol (2017) 8:968. 10.3389/fimmu.2017.00968 28848561 PMC5554366

[62] Abebe F Belay M Legesse M Mihret A Franklin KS Association of ESAT-6/CFP-10-induced IFN-gamma, TNF-alpha and IL-10 with clinical tuberculosis: evidence from cohorts of pulmonary tuberculosis patients, household contacts, and community controls in an endemic setting. Clin Exp Immunol (2017) 189(2):241–9. 10.1111/cei.12972 28374535 PMC5508323

