# Supplementary material for: Anomalies in T Cell Function Are Associated With Individuals at Risk of Mycobacterium abscessus Complex Infection
Source: Front Immunol. 2018 Jun 11;9:1319. doi: 10.3389/fimmu.2018.01319 (PMC6004551; doi:10.3389/fimmu.2018.01319)
Supplement: Supplementary file 1 [file data_sheet_1.PDF]

Supplementary Figure 1

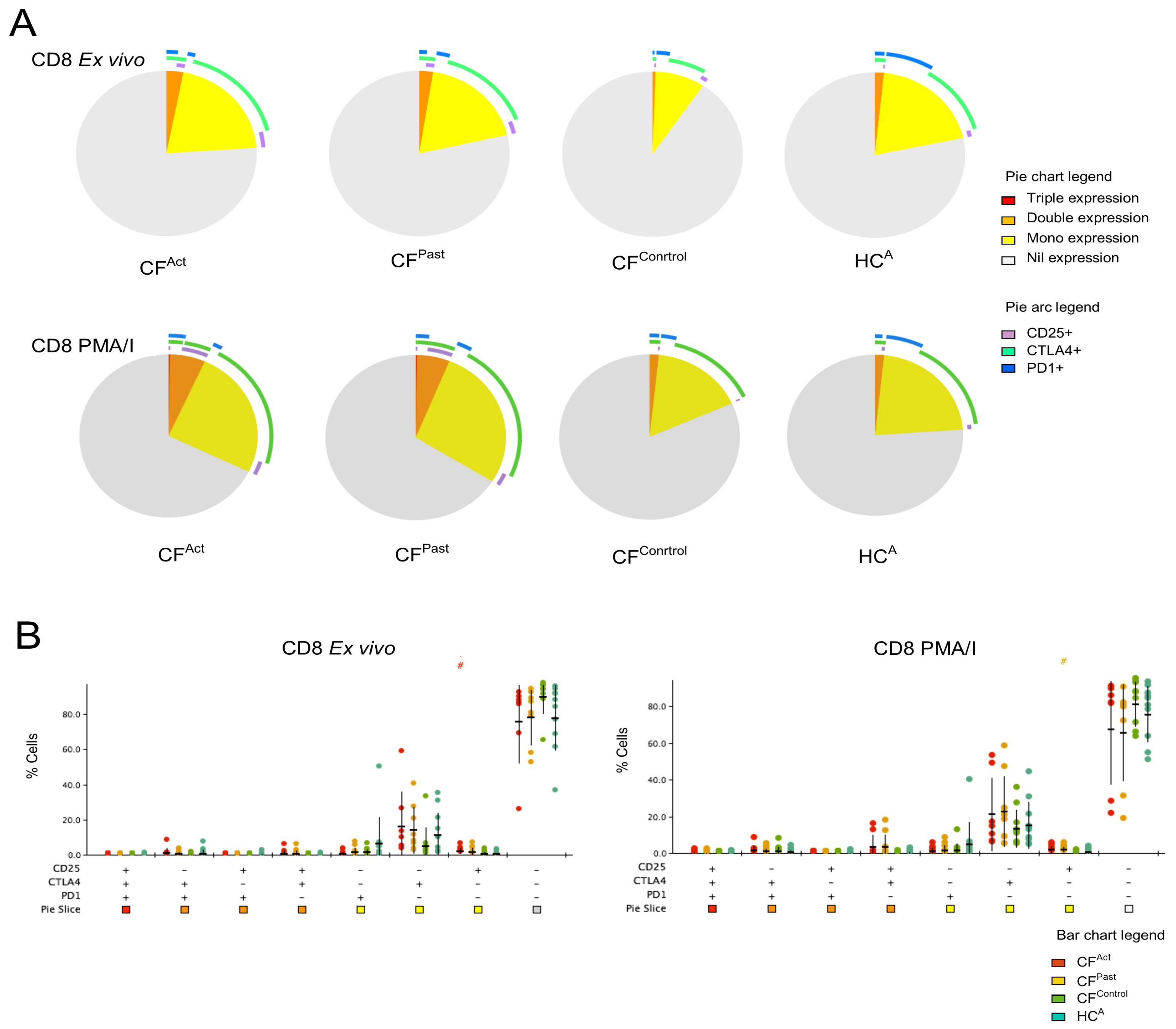

Supplementary Figure 1. **Immune activation and exhaustion marker profile in CD8$^+$ T cells in CF patient and control cohorts**. Flow cytometric phenotype profiling of CD8$^+$ T cells by SPICE showed no significant differences between CF^{Act}, CF^{Past}, CF^{Control} and HC^A groups directly *ex vivo* and post PMA/I stimulation. (**A**) Pie charts showing marker fingerprint on CD8$^+$ T cells before and after PMA/I stimulation. (**B**) Dot plots are shown showing all combinations of marker expression on CD8$^+$ T cells before and after PMA/I stimulation. Groups with significantly different expression compared to HC^A (Wilcoxon rank test $p<0.05$) are indicated by # symbol. Increased expression of CD25 on CD8$^+$ T cells is seen in NTM infection.
