# Supplementary material for: Anomalies in T Cell Function Are Associated With Individuals at Risk of Mycobacterium abscessus Complex Infection
Source: Front Immunol. 2018 Jun 11;9:1319. doi: 10.3389/fimmu.2018.01319 (PMC6004551; doi:10.3389/fimmu.2018.01319)
Supplement: Supplementary file 2 [file data_sheet_2.PDF]

Supplementary Figure 2

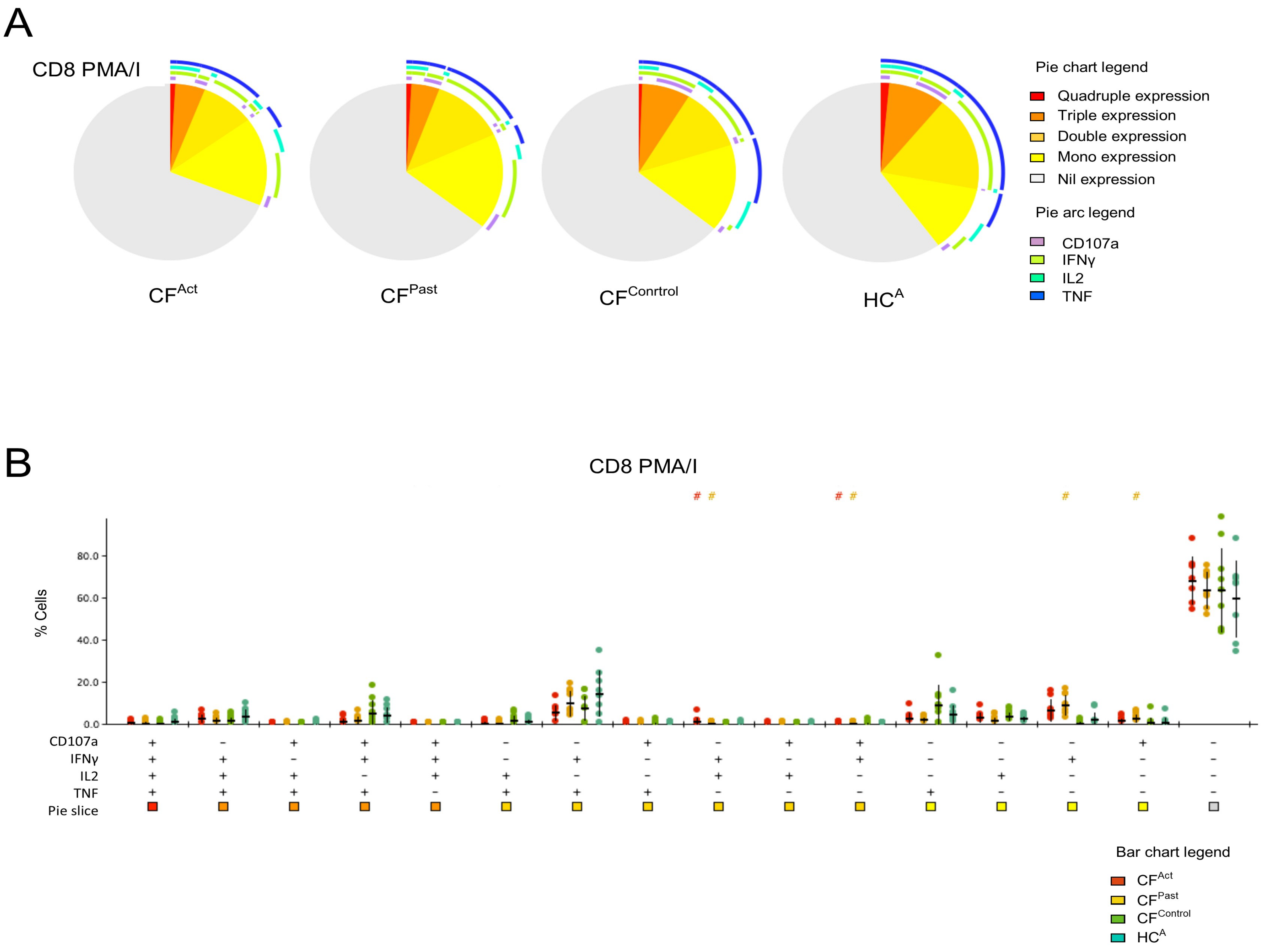

Supplementary Figure 2. **Polyfunctionality profile of CD8+ T cells post mitogen stimulation**. Flow cytometric phenotype profiling of CD8+ T cells post PMA/I stimulation by SPICE showed no significant differences between CF^Act, CF^Past, CF^Control and HC^A groups overall. (**A**) Pie charts showing cytokine polyfunctionality fingerprint of CD8+ T cells. (**B**) Dot plots show cytokine secretion profile in CD8+ T cells after PMA/I stimulation. Groups significantly different to HC^A are indicated with # symbol. Colour indicates significantly different group. Significantly higher interferon gamma secreting cells are seen as shown in Figure 2A.
