# Supplementary material for: Anomalies in T Cell Function Are Associated With Individuals at Risk of Mycobacterium abscessus Complex Infection
Source: Front Immunol. 2018 Jun 11;9:1319. doi: 10.3389/fimmu.2018.01319 (PMC6004551; doi:10.3389/fimmu.2018.01319)
Supplement: Supplementary file 3 [file data_sheet_3.PDF]

Supplementary Figure 3

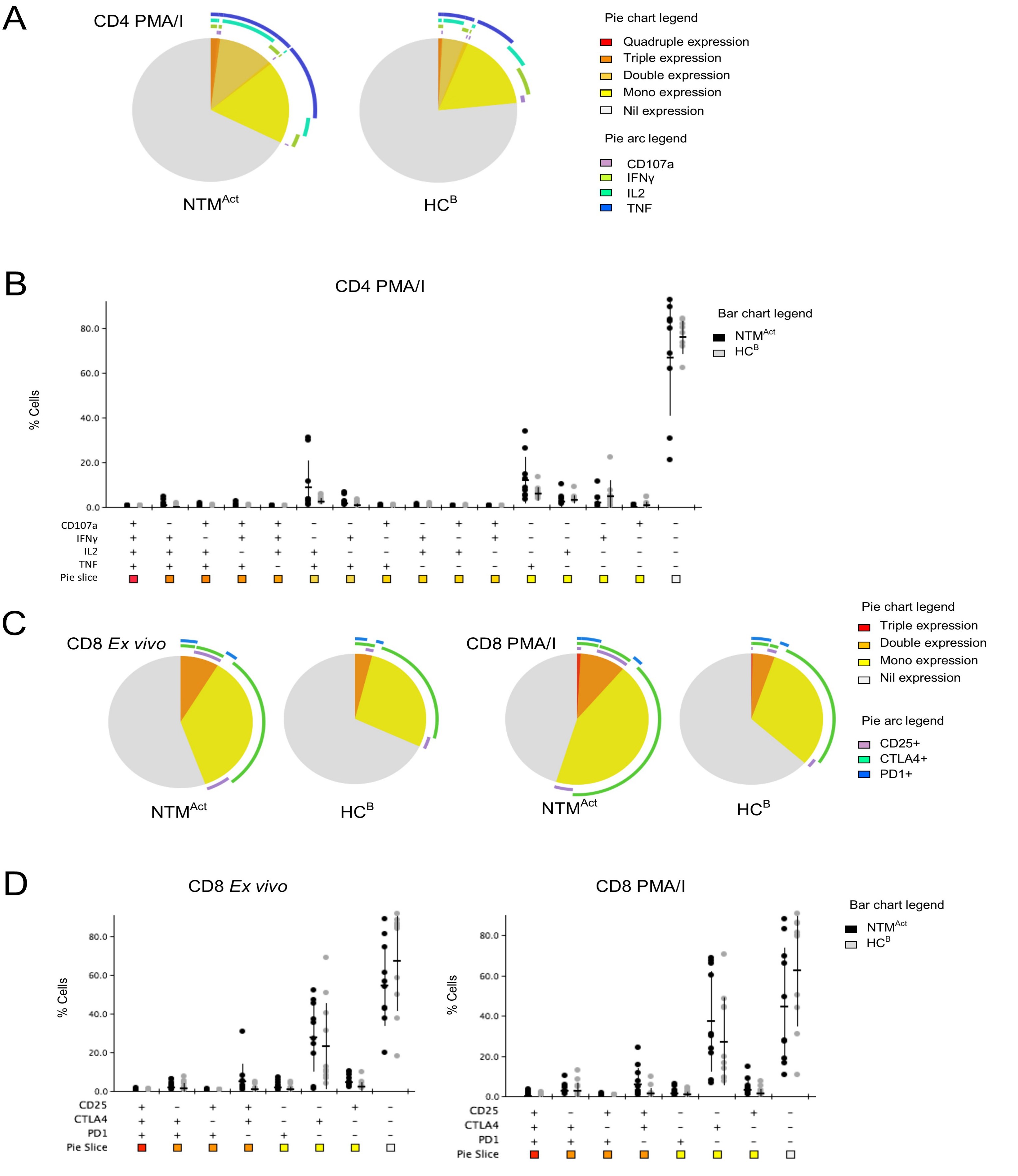

Supplementary Figure 3. **Phenotype and polyfunctionality profiles of T cells in immunocompetent individuals with active NTM infection.** (**A**) Flow cytometric polyfunctionality profiling of PMA/I stimulated $CD4^+$ T cells showed no significant differences between NTM^{Act} and HC^B groups. (**B**) SPICE dot plots showed no significant differences between NTM^{Act} and HC^B groups. (**C**) Flow cytometric phenotyping of *ex vivo* PMA/I stimulated $CD8^+$ T cells showed no significant differences between NTM^{Act} and HC^B groups. (**D**) SPICE dot plots showed no significant differences between NTM^{Act} and HC^B groups.
